# Posterior shoulder dislocation with associated reverse Hill–Sachs lesion: clinical outcome 10 years after joint-preserving surgery

**DOI:** 10.1007/s00402-022-04482-6

**Published:** 2022-06-03

**Authors:** Christian Konrads, Marie I. Konrads, Stefan Döbele, Tina Histing, Patrick Ziegler

**Affiliations:** 1 Department for Trauma and Reconstructive Surgery, BG Klinik, University of Tübingen, Schnarrenbergstr. 95, 72076 Tübingen, Germany; 2 Present Address: Department of Orthopaedic Surgery, University Hospital Tübingen, University of Tübingen, Hoppe-Seyler-Str. 3, 72076 Tübingen, Germany

**Keywords:** Glenohumeral instability, Proximal humerus fracture, Shoulder arthritis, Malgaigne lesion, McLaughlin lesion

## Abstract

**Introduction:**

Posterior shoulder dislocation in association with reverse Hill–Sachs lesion is a rather rare injury. Few studies reporting results after joint-preserving surgery in these cases are available. This current study presents the clinical outcomes 10 years postoperatively.

**Materials and methods:**

In a prospective case series, we operatively treated 12 consecutive patients (all males) after posterior shoulder dislocation with associated reverse Hill–Sachs lesion using joint-preserving techniques. Patients received surgery in a single center between January 2008 and December 2011. The joint-preserving surgical procedure was chosen depending on the defect size and bone quality. The following outcome-measures were analyzed: Constant-Score, DASH-Score, ROWE-Score, and SF-12. Results 1, 5, and 10 years postoperatively were compared.

**Results:**

Out of 12 patients, ten patients (83.3%) were followed-up with a mean follow-up interval of 10.7 years (range 9.3–12.8). The mean patient age at the time of the last follow-up was 51 years (32–66). The outcome scores at the final follow-up were: Constant 92.5 (range 70.0–100), DASH 3.2 (0.0–10.8), ROWE 91.0 (85.0–100), and SF-12 87.8 (77.5–98.3). Clinical results had improved from 1 to 5 years postoperatively and showed a tendency for even further improvement after 10 years.

**Conclusions:**

Joint-preserving surgical therapy of posterior shoulder dislocation provides excellent results when the morphology of the reverse Hill–Sachs lesion is respected in surgical decision-making.

**Trial registration:**

223/2012BO2, 02 August 2010.

## Introduction

Anterior shoulder dislocation is a common injury. Posterior shoulder dislocation is found in only 2% to 5% of all shoulder dislocations [1]. Possible reasons for posterior glenohumeral dislocations are seizure, electric shock, direct or indirect trauma that occurs with flexion, adduction, and internal rotation of the shoulder [1].

**Fig. 1:**
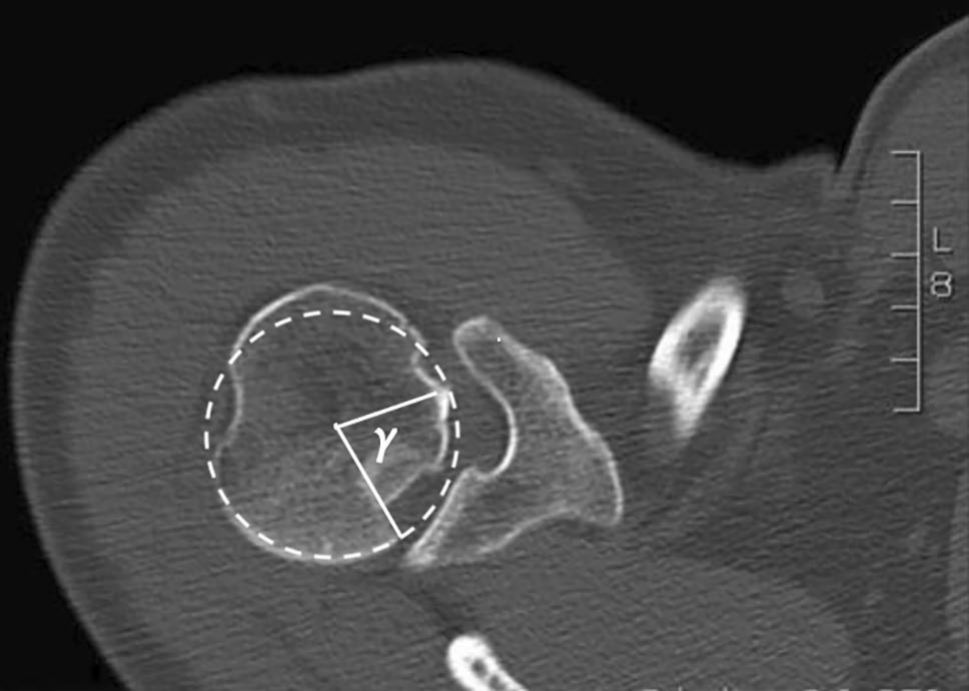
Axial CT slide of a right shoulder after posterior dislocation showing a reverse Hill–Sachs lesion. The gamma-angle (γ) is measured between the posterior defect edge and the bicipital groove after a best-fit circle was placed over the remainder of the humeral articular surface

Conservative management is possible in a stable situation after closed reduction, if there is no significant bone defect. Depending on the defect size and location, the reverse Hill–Sachs lesion is an important risk factor for re-dislocation and often requires surgical treatment [2]. The so-called gamma-angle (Fig. 1) can be measured on axial CT slide between the posterior defect edge and the bicipital groove [3]. Engagement of the reverse Hill–Sachs lesion with the posterior glenoid rim is more likely the larger the measured gamma-angle is [4].

For addressing the defect at the anterior part of the humeral head, a variety of surgical procedures exist: (a) filling the defect by subscapularis tendon transposition [5], (b) medial transposition of the lesser tuberosity [6, 7], (c) defect-filling with allograft [8], (d) rotational osteotomy [9], (e) arthroplasty [10, 11], and (f) arthroscopic or open elevation and stabilization of the impression fracture [12].

The aim of the present study was to analyze the long-term clinical outcome after joint-preserving surgery of posterior shoulder dislocation associated with reverse Hill–Sachs lesion.

## Materials and methods

**Fig. 2:**
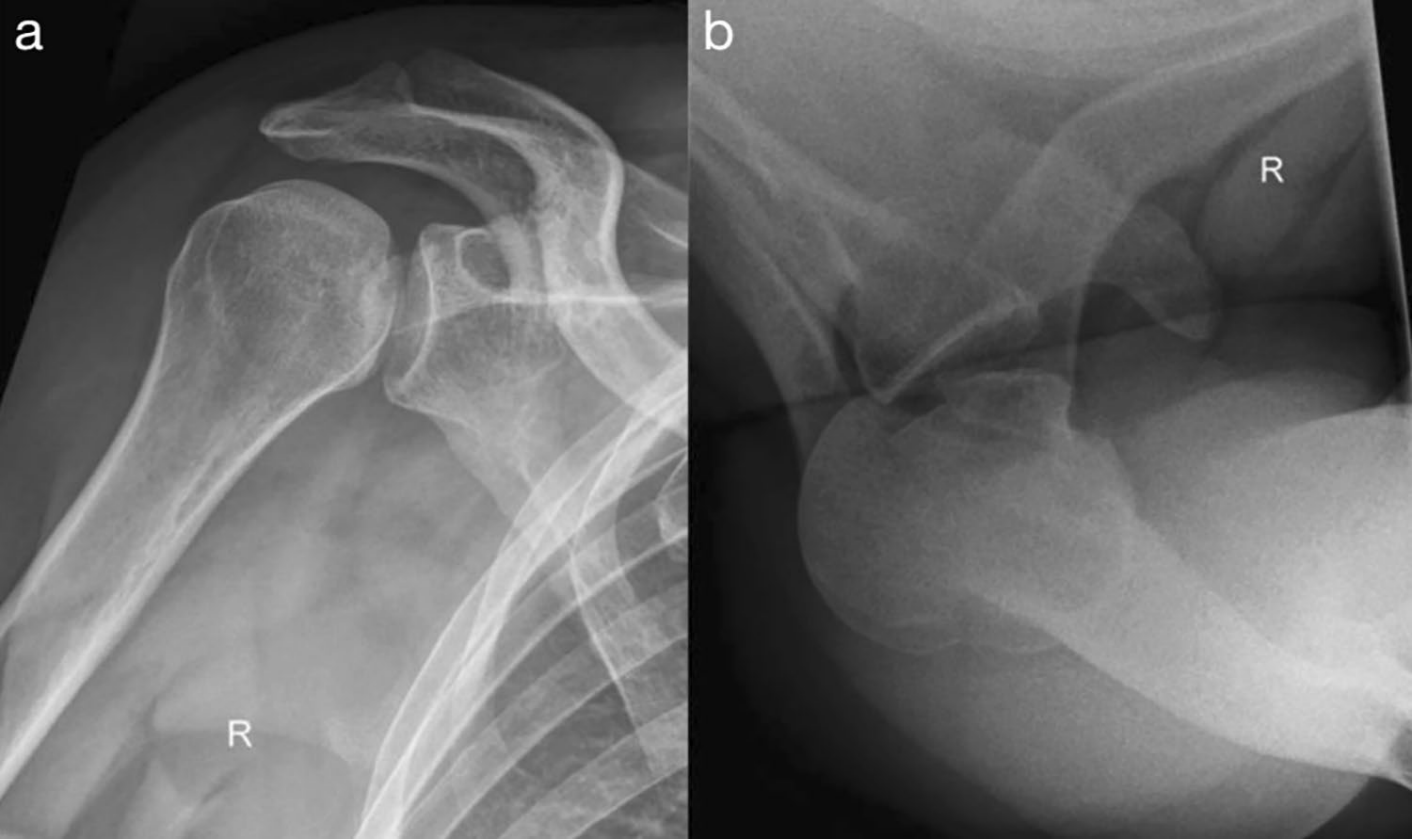
Radiographs of a posterior glenohumeral dislocation in a right shoulder showing a reverse Hill–Sachs lesion. **a** ap-view and **b** axial view

Between January 2008 and December 2011, 17 patients were treated at a level I trauma center because of posterior shoulder dislocation with associated reverse Hill–Sachs lesion. The diagnosis was confirmed using conventional radiography: true ap-view and axial view (Fig. 2). Four patients could be treated non-operatively. Closed reduction was accomplished using the method of Cicak [13]. One patient received arthroplasty. These five patients were excluded from the study. All of the conservatively managed patients had a Hill–Sachs defect size of less than 25% of the articular surface and the glenohumeral joint was stable. The patient who received a reversed total shoulder prosthesis had a defect size of greater than 40%. The remaining 12 patients had a reverse Hill–Sachs compression fracture involving 25–40% of the articular surface of the humeral head. All patients received a CT scan of the affected shoulder.

In this prospective case series, two surgeons operatively treated 12 patients after posterior shoulder dislocation with associated reverse Hill–Sachs lesion using joint-preserving techniques. All patients were male with a mean age of 39 (20–55) years at the time of injury. The surgical procedure was chosen depending on the defect size and bone quality.

For surgical treatment, the patient was positioned in beach-chair position under general anesthesia. All surgeries started with diagnostic arthroscopy. The depth of the bone defect and the glenohumeral cartilage were visualized. If no deep lesions of the cartilage worse than grade 2 according to the ICRS classification were detected and the time between the shoulder dislocation and the operative treatment was less than 14 days, the joint surface was restored by retrograde elevation of the impression fracture using a target device from the cruciate ligament instrumentation set under arthroscopic visualization.

**Fig. 3:**
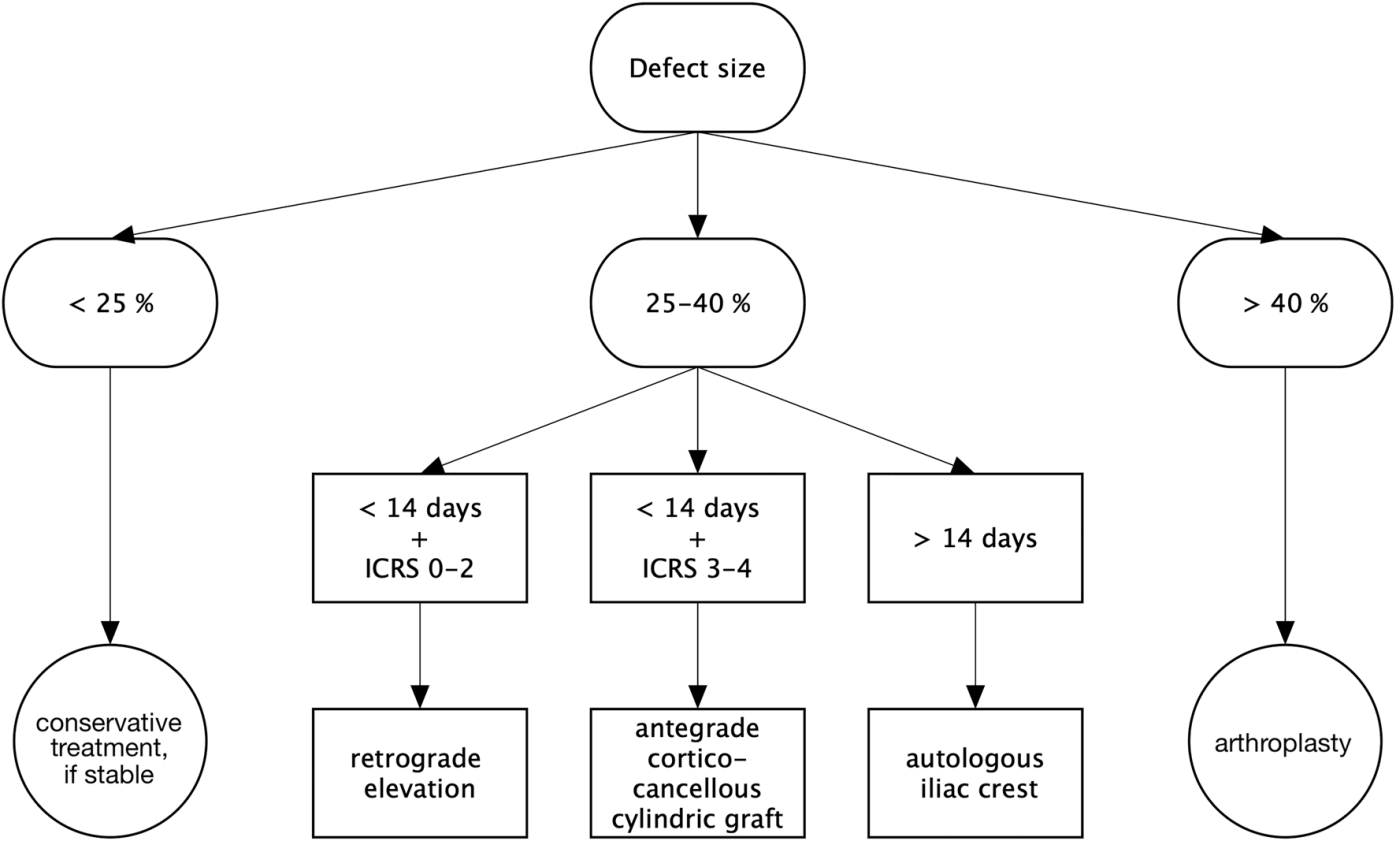
Treatment algorithm for posterior shoulder dislocation depending on the size of a reverse Hill–Sachs lesion and the time between trauma and surgery

In cases with cartilage lesions ICRS grade 3 or 4, open debridement using a deltopectoral approach was performed. If the time between the shoulder dislocation and the surgery was less than 14 days, the joint surface was reconstructed using antegrade cortico-cancellous cylindrical grafts from the iliac crest. If the surgery was performed later than 14 days after shoulder dislocation, the defect was reconstructed using a part of the autologous iliac crest fixed by small fragment screws. In all cases, it was of utmost importance to reconstruct the joint surface of the proximal humerus in a centered and stable glenohumeral joint. Figure 3 shows the different procedures used and the reasoning for indicating those treatment strategies.

The postoperative rehabilitation protocol included a standard shoulder abduction orthosis and assisted movement until 80° of abduction and flexion for 4 weeks. Internal rotation behind the back was not allowed until 6 weeks after surgery. In open approaches involving the subscapularis insertion, no external rotation was allowed for 4 weeks. Training of the active shoulder stabilizers was started 6 weeks after surgery.

All follow-up examinations were conducted by interviewing and examining the patients in person. For the Constant-Score, the Mecmesin Myometer was used. The following outcome measures were analyzed: Constant-Score [14, 15], DASH-Score [16], ROWE-Score [17], and SF-12. The subjective perception of pain was evaluated using an analogue scale (0–100). Results 1, 5, and 10 years postoperatively were compared.

Statistical analysis was performed using SPSS (version 22.0, SPSS Inc., Chicago, USA). The *t*-test was used to calculate differences between 1-, 5-, and 10-year evaluations of pain and function. Differences in outcome between the surgical techniques were calculated using the Kruskal–Wallis variance method. A p-value smaller than 0.05 was considered significant.

The trial had been registered at the German registry of clinical studies under the number 223/2012BO2 on 02 August 2010. Informed consent was obtained from all individual participants included in the study. The study was conducted according to the Helsinki Declaration (Ethical principles for medical research involving human subjects) and was approved by the ethics committee of the University of Tübingen, Tübingen, Germany.

## Results

Out of 12 patients, ten patients (83.3%) were followed-up with a mean follow-up interval of 10.7 years (range 9.3–12.8). The number one cause for posterior shoulder dislocation was high energy trauma. One patient had a bilateral dislocation due to an epileptic seizure. One side was treated surgically, and the other side could successfully be treated conservatively.

The injury to the cartilage of the humeral head was graded according to ICRS as grade 0–2 in 42% of all cases. These patients were treated by arthroscopically assisted elevation of the articular surface. 43% of the cases had chondral injuries grade 3 or higher according to ICRS and were treated using antegrade cortico-cancellous cylindrical grafts. Three polytrauma patients, who had been treated more than 14 days after injury, received cortico-cancellous bone grafts from their iliac crest. Diagnostic arthroscopy, which was performed in all cases at the beginning of the operation, revealed two posterior glenohumeral labral lesions but no rotator cuff lesions. The two labral lesions were addressed using arthroscopic suture anchor refixation.

There were not any intraoperative nerve injuries, postoperative infections, or other complications like hematoma or re-dislocation. The average length of in-hospital stay was 7.6 (4–24) days. An in-hospital stay of 24 days was reached by a polytrauma patient.

The mean patient age at the time of the last follow-up was 51 years (32–66). The outcome scores at the final follow-up were: Constant 92.5 (70.0–100), DASH 3.2 (0.0–10.8), ROWE 91.0 (85.0–100), and SF-12 87.8 (77.5–98.3). Clinical results had improved from 1 to 5 years postoperatively and showed a tendency for even further improvement after 10 years (Table 1).

**Table 1:** Clinical outcome for short-, mid-, and long-term follow-up after joint-preserving surgery for posterior shoulder dislocation with associated Hill–Sachs lesion

Score	2010	2015	2021
Constant	81.9 ± 3.1	89.5 ± 2.3	92.5 ± 8.9
ROWE	72.9 ± 5.6	87.9 ± 3.6	91.0 ± 7.4
DASH	10.5 ± 2.6	5.2 ± 1.4	3.2 ± 3.9
SF-12	Not evaluated	Not evaluated	87.8 ± 6.7
VAS	1.7 ± 0.4	0.8 ± 0.2	1.9 ± 3.8

The size of the reverse Hill–Sachs lesion did not have a significant impact on clinical outcome after surgery (*p* > 0.05). Also, in this limited cohort of patients, no significant difference in clinical outcome was found between open or arthroscopic procedures (*p* > 0.05).

## Discussion

In cases of joint-preserving surgery after posterior shoulder dislocation with associated reverse Hill–Sachs lesion, the study demonstrated excellent clinical long-term outcome. Patients have been improving in regards of objective shoulder function and subjective satisfaction between 1 and 5 years postoperatively. This implicates that healing and adaptation processes are usually not finished 1 year after surgery. Even between 2015 and 2021, there was still a tendency for further improvement of subjective results and objective shoulder function (*p* < 0.005). This implicates that there is no symptomatic arthritis or recurrent glenohumeral instability 10 years after surgery (Table 1).

According to our results and the current literature, the surgical techniques utilized for this study are powerful tools to restore shoulder function and quality of life [18]. The presented results are comparable or even better than previously published data from other study groups [18]. Reverse Hill–Sachs lesions smaller than 25% with stable shoulder joint situations should be treated non-operatively using an external rotation orthosis for 4–6 weeks. In these cases, good-to-excellent clinical outcome 5 years after injury can be expected [19, 20].

The treatment algorithm used for the present study proved to be helpful with satisfying short- and long-term outcomes (Fig. 3). However, a new classification for posterior shoulder dislocations associated with reverse Hill–Sachs lesion might facilitate the decision process when indicating conservative or surgical treatment. Such a classification or algorithm could include the gamma-angle (Fig. 1) and maybe the defect depth [3, 4, 20]. This would require a multi-center study using a high amount of clinical and radiological data.

The main limitation of this study is the small number of cases. It would be interesting to analyze the possible development of posttraumatic glenohumeral arthritis, but evaluation of long-term follow-up radiographs was not possible in the context of this study, because this would require a certain amount of X-ray exposure for scientific interest without clinical consequence. Therefore, long-term follow-up radiographic examination was not part of the approved study protocol.

Open and arthroscopic procedures cannot be compared directly in the setting of this study due to differences in injury severity and indications (Fig. 3). Statistical analysis of different subgroups (e.g., defect size of the reverse Hill–Sachs lesion) was underpowered because of the small patient cohort. However, reconstruction of anatomy seems to successfully restore shoulder function in the examined group of patients.

## Conclusions

Joint-preserving surgical therapy of posterior shoulder dislocation provides excellent results when the morphology of the reverse Hill–Sachs lesion is respected in surgical decision-making. Open and minimally invasive techniques demonstrated promising subjective and objective outcomes in the respected indications. Reconstruction of the joint surface morphology and glenohumeral stability most likely results in excellent long-term clinical outcome.

## Data Availability

The datasets used and analyzed during the current study are available from the corresponding author on reasonable request.

## Abbreviations

CT: Computed tomography
DASH: Disability of the arm, shoulder, hand
ICRS: International cartilage repair society
MRI: Magnetic resonance imaging
VAS: Visual analogue scale
